# Two decades of nutrition research in polycystic ovary syndrome: emerging patterns and scientific output (2004–2024)

**DOI:** 10.3389/fnut.2026.1763141

**Published:** 2026-05-14

**Authors:** Mevra Aydin Cil, Elif Ulug

**Affiliations:** Department of Nutrition and Dietetics, Faculty of Health Sciences, Ataturk University, Erzurum, Türkiye

**Keywords:** bibliometric analysis, diet, endocrine diseases, nutrition, polycystic ovary syndrome

## Abstract

**Objective:**

Polycystic ovary syndrome (PCOS) is a complex endocrine disorder with significant metabolic and reproductive effects. Although various nutritional strategies have been studied, an overarching review of nutrition-focused research in PCOS remains limited. This study aimed to present a comprehensive bibliometric analysis of this research area.

**Methods:**

A bibliometric analysis was conducted using publications from the Web of Science and Scopus databases from 2004 to 2024. Two independent reviewers screened the studies. Bibliometric indicators and network visualizations were analyzed via Microsoft Excel, the *bibliometrix* package R, VOSviewer, and CiteSpace.

**Results:**

A total of 1,248 publications met the inclusion criteria. The United States, Iran, and China were the top contributing countries. Notable authors with strong collaborative networks included Teede HJ, Escobar-Morreale HF, and Moran LJ. Keyword co-occurrence analysis identified four primary thematic clusters: lifestyle modifications, hormonal/fertility-related issues, metabolic disturbances, and dietary interventions. Burst analysis and timeline mapping have led to a growing research focus on emerging topics such as “gut microbiota,” “ketogenic diet,” and “oxidative stress.”

**Conclusion:**

This bibliometric analysis provides a comprehensive overview of two decades of nutrition-related PCOS research. These highlights increasing scientific interest in dietary strategies and highlights key emerging themes, such as the gut microbiota, ketogenic diets, and oxidative stress.

## 1 Introduction

Polycystic ovary syndrome (PCOS) is among the most prevalent endocrine disorders in women of reproductive age, affecting approximately one in five women. This syndrome is clinically characterized by androgen excess/hyperandrogenism, irregular menstruation, and polycystic ovarian morphology (1). In addition to reproductive complications, PCOS is associated with several metabolic and systemic consequences (2, 3). At this point, PCOS is associated with an increased risk for developing reproductive, metabolic, cardiovascular, oncological, and psychological disturbances (2–4). Meta-analyses have revealed that women with PCOS exhibit a greater prevalence of cardiometabolic risk factors that increase long-term healthcare costs, including metabolic syndrome, type 2 diabetes mellitus, dyslipidemia/hyperlipidemia, hypertension, and chronic low-grade inflammation (5, 6). These findings highlight the need for early diagnosis and comprehensive, multidisciplinary management strategies to mitigate the long-term health burden associated with PCOS.

Nutrition represents a modifiable risk factor for metabolic disorders, and medical nutrition therapy constitutes an important component of multidisciplinary management of metabolic disorders (7, 8). In this context, nutritional regulation serves as a highly effective therapeutic strategy—complementing appropriate pharmacological interventions—for the prevention and management of cardiometabolic risk factors associated with PCOS (1, 9, 10). A growing body of research has investigated the potential therapeutic impacts of various dietary patterns, such as the Mediterranean diet (11), low-glycemic index diets (12), the ketogenic diet (13), and time-restricted feeding regimens (14). In addition, numerous studies have revealed the roles of specific nutrients, such as different fatty acids (15), some vitamins and minerals (e.g., vitamin D, zinc, and magnesium) (16, 17), vitamin-like molecules (e.g., myo-inositol) (17), and phytochemicals (e.g., catechins and polyphenols) (16, 18), in modulating insulin sensitivity, oxidative stress, inflammation, and hormonal imbalance—key pathophysiological mechanisms implicated in PCOS. Despite these promising results, the current literature remains inconclusive, and there is no universally accepted medical nutrition therapy for the optimal management of PCOS.

Given the abundance of research and the persistent lack of consensus regarding effective dietary strategies for the management of PCOS, there is a pressing need to comprehensively map the current scientific status in this field. In this context, bibliometric analysis offers a valuable tool for systematically assessing publication trends, thematic focus areas, leading authors, collaborative networks, and existing research gaps within the literature. Accordingly, the present study aims to conduct a comprehensive bibliometric analysis of nutrition-related research in the context of PCOS, with the objective of identifying the most extensively investigated dietary approaches, highlighting emerging scientific themes, and guiding future research toward the development of evidence-based nutritional interventions for PCOS management.

## 2 Materials and methods

### 2.1 Data collection and search strategy

A literature search was conducted using the Web of Science (WoS) and Scopus databases on nutrition in PCOS patients. The search was conducted in the Web of Science database via the following search query: (PCOS OR “polycystic ovary syndrome”) AND (nutrition OR diet* OR “diet therapy” OR “nutritional assessment” OR nutrient) within the topic field; and in the Scopus database via the following search query: (PCOS or “polycystic ovary syndrome”) AND (nutrition or diet* or “diet therapy” or “nutritional assessment” or nutrient) within the article title, abstract, and keywords field on 10 March 2025The literature review encompassed a 20-year period from 2004 to 2024 and included only articles published in English. Publications classified as meeting abstracts, books, book chapters, meetings, proceeding papers, letters, corrections, retracted publications, expressions of concern, notes, short surveys, and errata were excluded from the final articles.

The publications were retrieved from the databases as full records, and duplicates were removed. Thereafter, two independent researchers assessed the publications to determine their relevance to PCOS and nutrition. Certain studies on PCOS were not focused on nutrition but were retrieved during the search due to the presence of terms such as diet modifications, nutrition, or lifestyle changes, which often appeared solely in the discussion and/or conclusion sections. However, as these studies did not primarily focus on nutrition nor include it as part of their research methodology, they were excluded from the bibliometric analysis. To ensure the relevance and specificity of the studies, the downloaded articles were independently assessed by two authors (EU and MAC), and studies that did not directly address PCOS and nutrition within their research scope were excluded from the data analysis.

### 2.2 Data analysis

To perform bibliometric analysis and visualization, Microsoft Excel, the *bibliometrix* package in RStudio, VOSviewer, and CiteSpace were used. Therefore, annual publication trends were analyzed and visualized in Microsoft Excel. Furthermore, for the creation of advanced networks and density visualizations, bibliographic data were prepared via the *bibliometrix* package in RStudio 4.4.3 and then transferred to Vosviewer (version 1.6.20) (19). In addition, the CiteSpace program (version 6.4. R2) was used for author collaboration and strongest citation burst analysis (20).

## 3 Results

**Figure 1:**
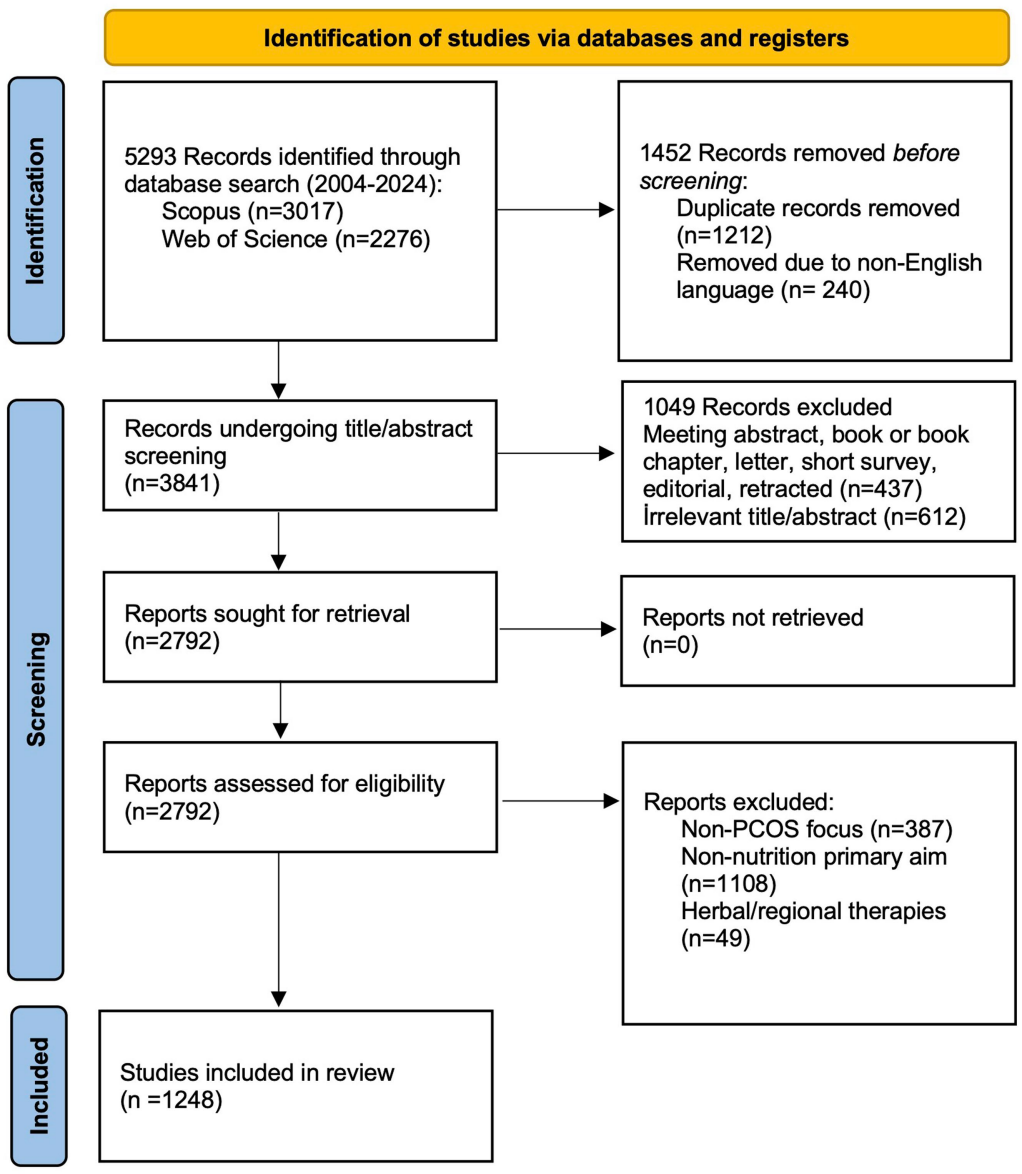
Flow diagram illustrating the identification, screening, eligibility assessment, and inclusion process of publications related to PCOS and nutrition retrieved from the Web of Science and Scopus databases between 2004 and 2024.

The flow chart regarding the search strategy and the processes of inclusion and exclusion of records is given in Figure 1. In the first search, 2,276 records were found in Web of Science, and 3,017 records were found in Scopus. Following the removal of duplicates and non-English language articles, 3,841 records were obtained. After applying the document type filter, a total of 2,792 records were identified. Afterwards, these records were independently evaluated by two authors (EU and MAC), and on the basis of the inclusion and exclusion criteria, 1,248 records were included for bibliometric analysis.

### 3.1 Annual publication trends

**Figure 2:**
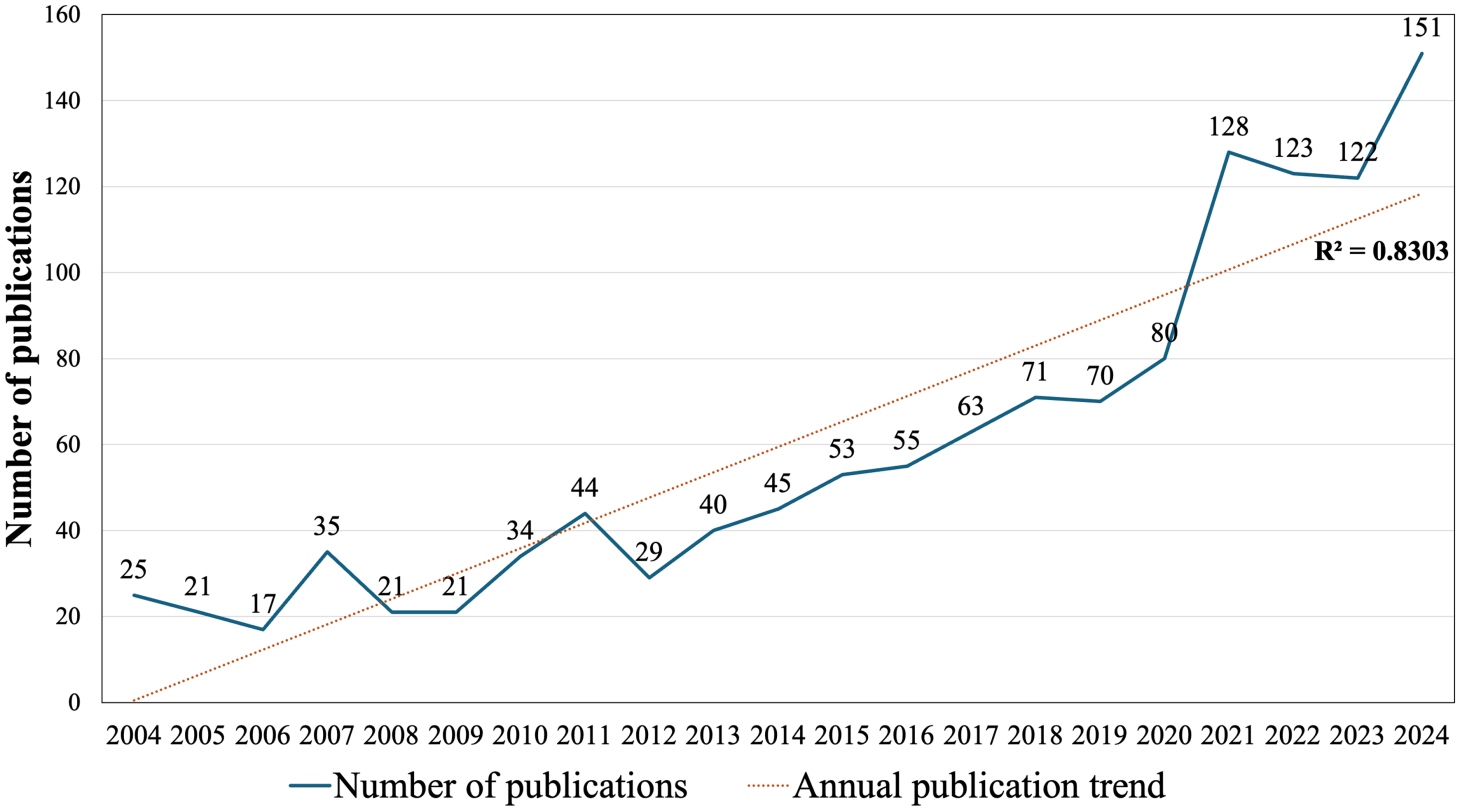
Annual publication trends in nutrition and PCOS research.

Although annual scientific output has fluctuated over the years, an increasing trend has been observed, particularly after 2012. The most substantial increase occurred between 2020 (80 publications) and 2021 (128 publications), reaching its highest point in 2024, with 151 publications. In this bibliometric analysis, the temporal distributions of publications were assessed via goodness-of-fit tests (21). A significant positive correlation between year and number of publications was identified, with a high coefficient of determination (R^2^ = 0.8303), as demonstrated by trendline analysis (Figure 2).

### 3.2 Countries and districts

The top 10 countries contributing to research on the association between nutrition and PCOS are summarized in Table 1. Fifty-three countries were identified as contributors to publications in this field. Among these, the United States, Iran, and China ranked as the top three publishing countries, collectively accounting for 50.4% of the total publications (630 out of 1,250). The United States presented the highest average number of citations per article (76.4), followed by Italy (65.1), Australia (51.1), the United Kingdom (35.9), and Canada (30.2; Table 1).

**Table 1:** Top 10 productive countries.

Rank	Country	Documents (n)	Citations (n)	Average citations (n)
1	United States	224	17,103	76.4
2	Iran	209	5,311	25.4
3	China	197	3,883	19.7
4	India	102	1,427	14.0
5	Italy	99	6,444	65.1
6	Australia	79	4,033	51.1
7	United Kingdom	58	2082	35.9
8	Brazil	39	742	19.0
9	Canada	37	1,117	30.2
10	Poland	37	697	18.8

### 3.3 Journals

A total of 543 different journals were published in the fields of PCOS and nutrition between 2004 and 2024. Table 2 presents detailed information on the top 10 journals with the highest number of publications in the PCOS and nutrition fields, including the number of publications in the journals, total citations, average citations per publication, the 2023 impact factor (IF), and the 5-year IF (Table 2). Accordingly, the journal *Nutrients* accounted for the highest number of publications in this field (54 publications, 4.32%). Furthermore, the *Journal of Clinical Endocrinology & Metabolism* ranked first in both total citations and average citations (4,142 and 125.5, respectively). In addition, the journal *Fertility and Sterility* presented the highest 2024 IFs and 5-year IFs among all the journals (7.0 and 7.1, respectively; Table 2).

**Table 2:** Top 10 journals with most publications on nutrition and PCOS.

Journal, publisher	Number of publications (n, %)	Total citations (n)	Average citations	IF (2024)	5-year IF
Nutrients, MDPI	54, 4.32%	1,471	27.2	5.0	6.0
Gynecological Endocrinology, Taylor & Francis	41, 3.28%	1,052	25.7	1.7	2.1
Journal of Clinical Endocrinology & Metabolism, Endocrine Society	33, 2.64%	4,142	125.5	5.1	5.4
Fertility and Sterility, Elsevier	29, 2.32%	1,424	49.1	7.0	7.1
Frontiers in Endocrinology, Frontiers	23, 1.84%	544	23.6	4.6	5.2
Clinical Endocrinology, Wiley -Blackwell Publishing	19, 1.52%	1789	94.2	2.4	2.9
Journal of Ovarian Research, BioMed Central	17, 1.36%	394	23.2	4.2	4.4
Human Reproduction, Oxford University Press	16, 1.28%	759	47.4	6.1	6.2
Biological Trace Element Research, Springer Nature	13, 1.04%	333	25.6	3.6	3.8
European Journal of Endocrinology, Oxford University Press	12, 0.96%	741	61.8	5.2	5.1

IF, Impact factor.

### 3.4 Citations

A total of 44,615 citations were made to the 1,248 articles included in the bibliometric analysis. Among the 1,248 publications, the top 10 publications ranked by total citations are listed in Table 3. The most cited article was published in Endocrine Reviews by Diamanti-Kandarakis E et al. (22) in 2009, with 3,185 citations, which was approximately twice as many as the second article (1,591 citations) (23). Furthermore, the third (24) and fourth (25) most cited publications received comparable numbers of citations (1,408 and 1,405 citations, respectively), whereas the fifth-ranked publication (26) had approximately half the citation count of these two with 713 citations (Table 3).

**Table 3:** Top 10 citation publications.

Rank	Authors	Number of citations	Titles	Journals
1	Diamanti-Kandarakis E (2009)	3,185	Endocrine-disrupting chemicals: an Endocrine Society scientific statement	Endocrine Reviews
2	Rochester J. R. (2013)	1,591	Bisphenol A and human health: a review of the literature	Reproductive Toxicology
3	Vander Borght, M (2018)	1,408	Fertility and infertility: Definition and epidemiology	Clinical Biochemistry
4	Legro RS (2013)	1,405	Diagnosis and treatment of polycystic ovary syndrome: an Endocrine Society clinical practice guideline	The Journal of Clinical Endocrinology and Metabolism
5	Teede HJ (2018)	713	Recommendations from the international evidence-based guideline for the assessment and management of polycystic ovary syndrome	Fertility and Sterility
6	Croze ML (2013)	411	Potential role and therapeutic interests of myo-inositol in metabolic diseases	Biochimie
7	Silvestris E (2018)	369	Obesity as disruptor of the female fertility	Reproductive Biology and Endocrinology
8	Legro RS (2004)	360	Detecting Insulin Resistance in Polycystic Ovary Syndrome: Purposes and Pitfalls	Obstetrical & Gynecological Survey
9	Escobar-Morreale, H. F., (2007)	320	Abdominal adiposity and the polycystic ovary syndrome	Trends in Endocrinology and Metabolism
10	Goodman NF (2015)	312	American association of clinical endocrinologists, American college of endocrinology, and androgen excess and pcos society disease state clinical review: Guide to the best practices in the evaluation and treatment of polycystic ovary syndrome	Endocrine Practice

### 3.5 Author collaboration analysis

**Figure 3:**
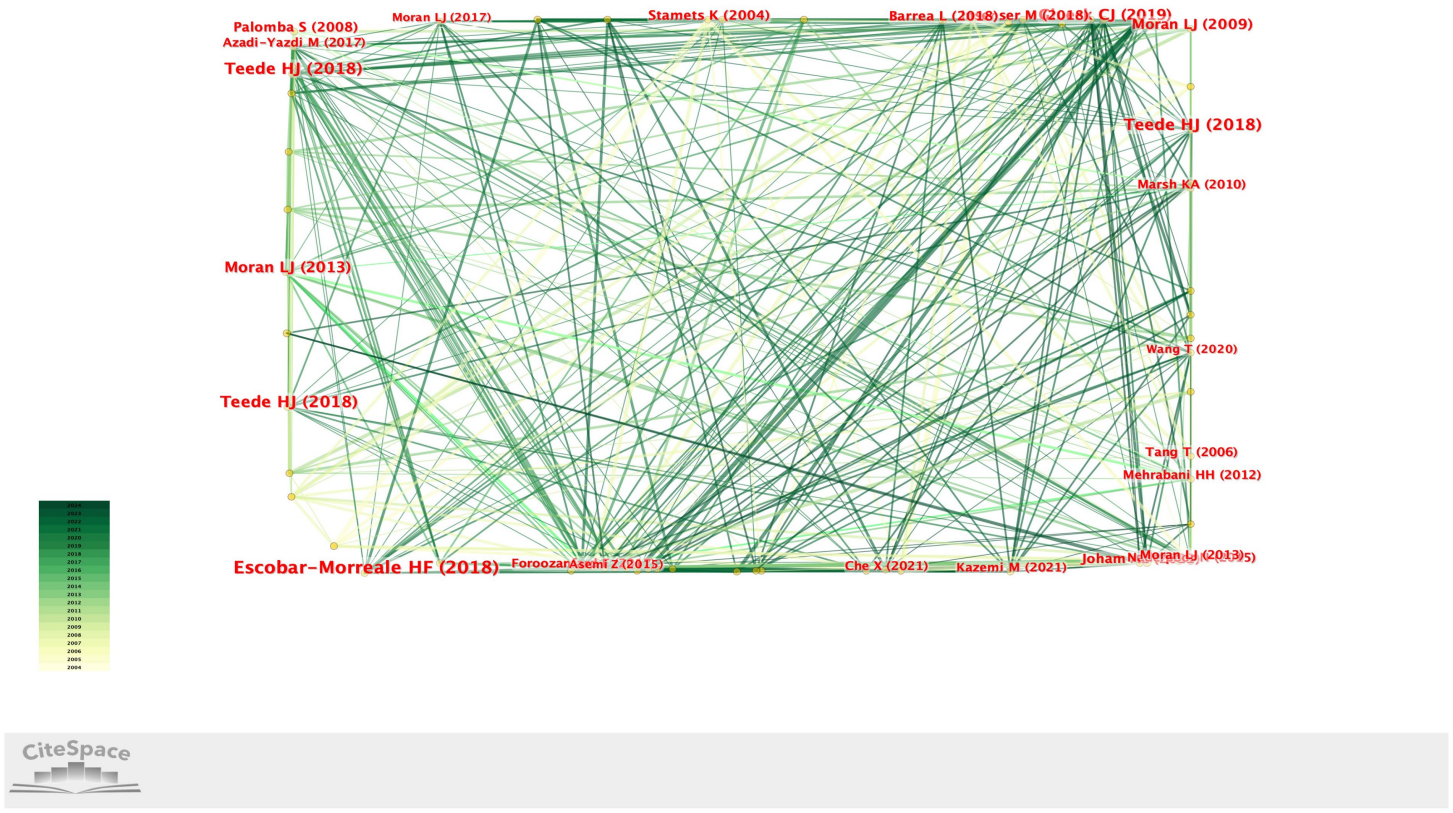
Author collaboration network map showing co-authorship relationships among researchers in the fields of nutrition and PCOS.

A total of 5,385 authors contributed to the relevant publications. For the author collaboration network analysis, only those with a publication frequency of 10 or more were included, resulting in the identification of 69 authors. The author collaboration network is illustrated in Figure 3. Specifically, Teede HJ (total link strength = 184), Escobar-Morreale HF (total link strength = 93), Moran LJ (total link strength = 81), and Glueck CJ (total link strength = 48) demonstrated the highest levels of collaboration, indicating strong research partnerships and recurrent scientific contributions within the field (Figure 3).

### 3.6 Co-citation distribution

**Figure 4:**
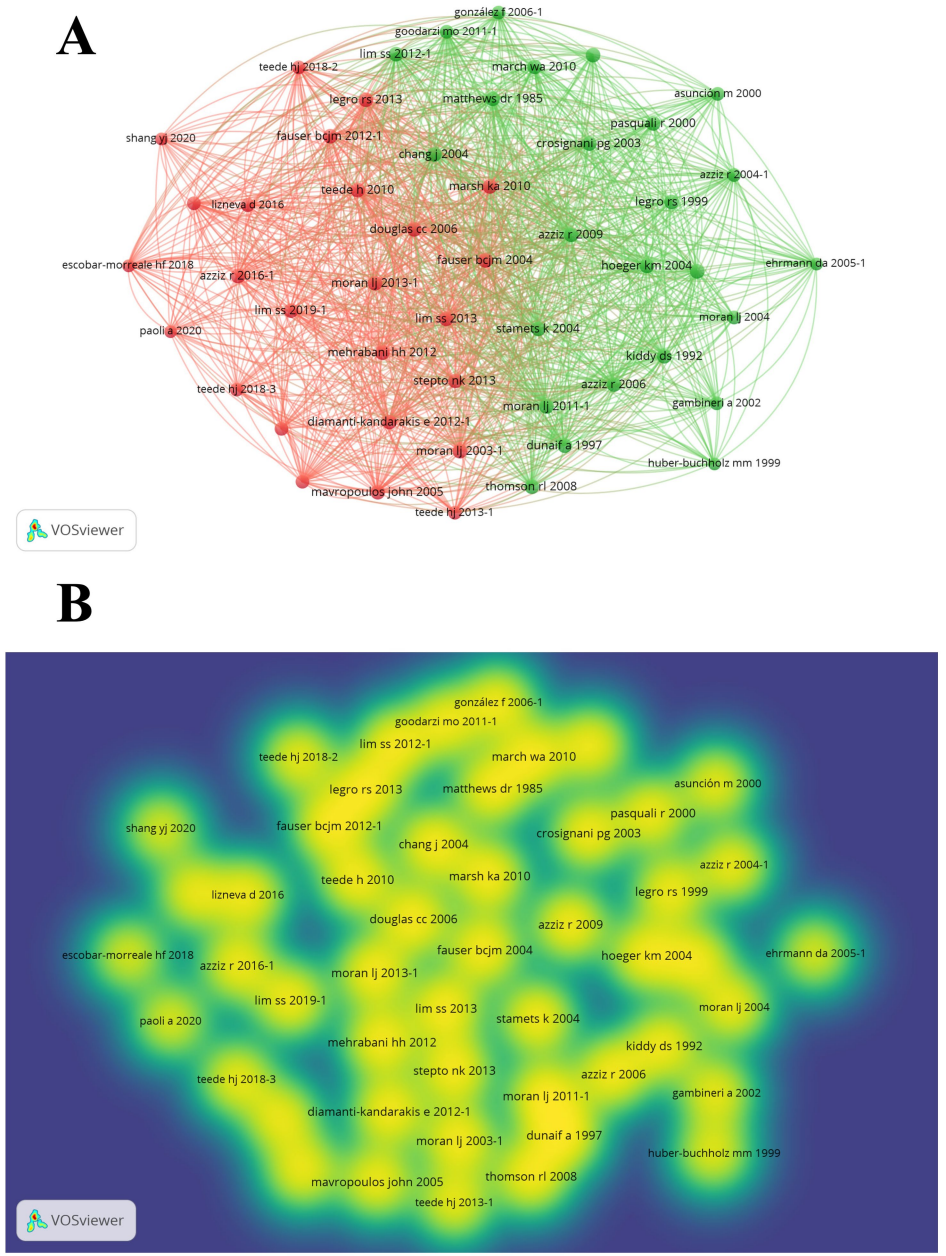
Co-citation analysis of cited references related to nutrition and PCOS. **(A)** Co-citation network of frequently cited articles. **(B)** Density visualization of co-cited literature.

Co-citation bibliometric analysis was performed on the 50 most cited articles, and network and density visualization graphs are presented in Figure 4. The size of the node in the density graph corresponds to the frequency of citations. Additionally, the authors and their manuscripts of the top 11 most highly co-cited articles are summarized in Table 4. Accordingly, the six publications presented identical co-citation frequencies, each receiving 49 co-citations (Table 4).

**Table 4:** Top 11 co-citations.

Rank	Authors	Number of co-citations	Titles	Journals
1	Rotterdam ESHRE/ASRM-Sponsored PCOS consensus workshop group (2004)	49	Revised 2003 consensus on diagnostic criteria and long-term health risks related to polycystic ovary syndrome	Fertility and Sterility
2	Douglass CC (2006)	49	Difference in dietary intake between women with polycystic ovary syndrome and healthy controls	Fertility and Sterility
3	Fauser BCJM (2012)	49	Consensus on women’s health aspects of polycystic ovary syndrome (PCOS): the Amsterdam ESHRE/ASRM-Sponsored 3rd PCOS Consensus Workshop Group	Fertility and Sterility
4	Marsh KA (2010)	49	Effect of a low glycemic index compared with a conventional healthy diet on polycystic ovary syndrome	The American Journal of Clinical Nutrition
5	Mehrabani HH (2012)	49	Beneficial effects of a high-protein, low-glycemic-load hypocaloric diet in overweight and obese women with polycystic ovary syndrome: a randomized controlled intervention study	Journal of the American College of Nutrition
6	Moran LJ (2013)	49	Dietary Composition in the Treatment of Polycystic Ovary Syndrome: A Systematic Review to Inform Evidence-Based Guidelines	Journal of the Academy of Nutrition and Dietetics
7	Legro RS (2013)	48	Diagnosis and treatment of polycystic ovary syndrome: an Endocrine Society clinical practice guideline	The Journal of Clinical Endocrinology and Metabolism
8	Moran LJ (2011)	48	Lifestyle changes in women with polycystic ovary syndrome	The Cochrane Database of Systematic Reviews
9	Azziz R (2006)	47	Positions statement: criteria for defining polycystic ovary syndrome as a predominantly hyperandrogenic syndrome: an Androgen Excess Society guideline	The Journal of Clinical Endocrinology and Metabolism
10	Teede H (2010)	47	Polycystic ovary syndrome: a complex condition with psychological, reproductive and metabolic manifestations that impacts on health across the lifespan	BMC Medicine
11	Thomson RL (2008)	47	The effect of a hypocaloric diet with and without exercise training on body composition, cardiometabolic risk profile, and reproductive function in overweight and obese women with polycystic ovary syndrome	The Journal of clinical endocrinology and metabolism

Authors with similar co-citations are listed in alphabetical order.

### 3.7 Keyword analysis

The top 50 keywords were identified from 2,221 keywords and were divided into 4 different clusters (red, green, blue, and yellow). Table 5 presents the keywords categorized by cluster, while Table 6 displays the top 10 keywords ranked by frequency of occurrence. Accordingly, “polycystic ovary syndrome (*n* = 2707)” was the most common keyword, followed by “insulin resistance (*n* = 1129)” and “PCOS (*n* = 1,079).” The frequencies of “obesity (*n* = 857)” and “diet (*n* = 638)” closely followed. Additionally, other keywords related to nutrition, such as “Vitamin D (*n* = 195)”, “lifestyle (*n* = 184)”, “nutrition (*n* = 173),” “myo-inositol (*n* = 105),” and “Mediterranean diet (*n* = 95)”, also appeared with relatively high frequencies (Table 6). Each cluster was defined based on the frequency of co-occurrence between keywords and the conceptual similarity of research topics using an automated clustering algorithm implemented in R, ensuring an objective data-driven classification. The clustering process was performed algorithmically, and the resulting groups were subsequently interpreted based on their thematic coherence, ensuring minimal overlap between clusters. The five most relevant words in the red cluster are “obesity,” “diet,” “inflammation,” and “syndrome.” In the green cluster, the top words are “hyperandrogenism,” “infertility,” “metformin,” and “fertility.” The most prominent words in the blue cluster are “insulin resistance,” “insulin,” “oxidative stress,” and “meta-analysis.” Finally, the five most relevant words in the yellow cluster are “PCOS,” “polycystic ovarian syndrome”, “lifestyle”, and “metabolic syndrome” (Table 5).

**Table 5:** Co-occurrence analysis of keywords in the 4 clusters.

Cluster	Color	Keywords (Occurrence)
Cluster 1	Red	obesity (44), diet (39), inflammation (30), syndrome (28), polycystic ovary (27), nutrition (25), polycystic ovary syndrome (24), weight loss (23), testosterone (19), quality of life (17), physical activity (17), overweight (17), depression (12), Mediterranean diet (12)
Cluster 2	Green	hyperandrogenism (35), infertility (29), metformin (26), fertility (25), anovulation (23), vitamin D (23), diabetes (23), inositol (19), myo-inositol (19), BMI (17), androgens (17), pregnancy (14)
Cluster 3	Blue	insulin resistance (45), insulin (32), oxidative stress (30), meta-analysis (27), systematic review (24), women (21), polycystic (19), glucose (19), gut microbiota (19), supplementation (16), lipid profile (16), ovary syndrome (15)
Cluster 4	Yellow	PCOS (44), polycystic ovarian syndrome (32), lifestyle (28), metabolic syndrome (28), exercise (21), resistance (16), ketogenic diet (15), ketogenic diet (15), hyperandrogenemia (14), adolescent (11), letrozole (11), high-fat diet (9)

**Table 6:** Ranking of keywords usage frequency.

Rank	Keywords	Cluster	Total link strength	Frequency
1	polycystic ovary syndrome	1	24	2,707
2	insulin resistance	3	45	1,129
3	PCOS	4	44	1,079
4	obesity	1	44	857
5	diet	1	39	638
6	hyperandrogenism	4	35	375
7	polycystic ovarian syndrome	4	32	353
8	Metabolic syndrome	4	28	331
9	infertility	2	29	312
10	metformin	2	26	301

**Figure 5:**
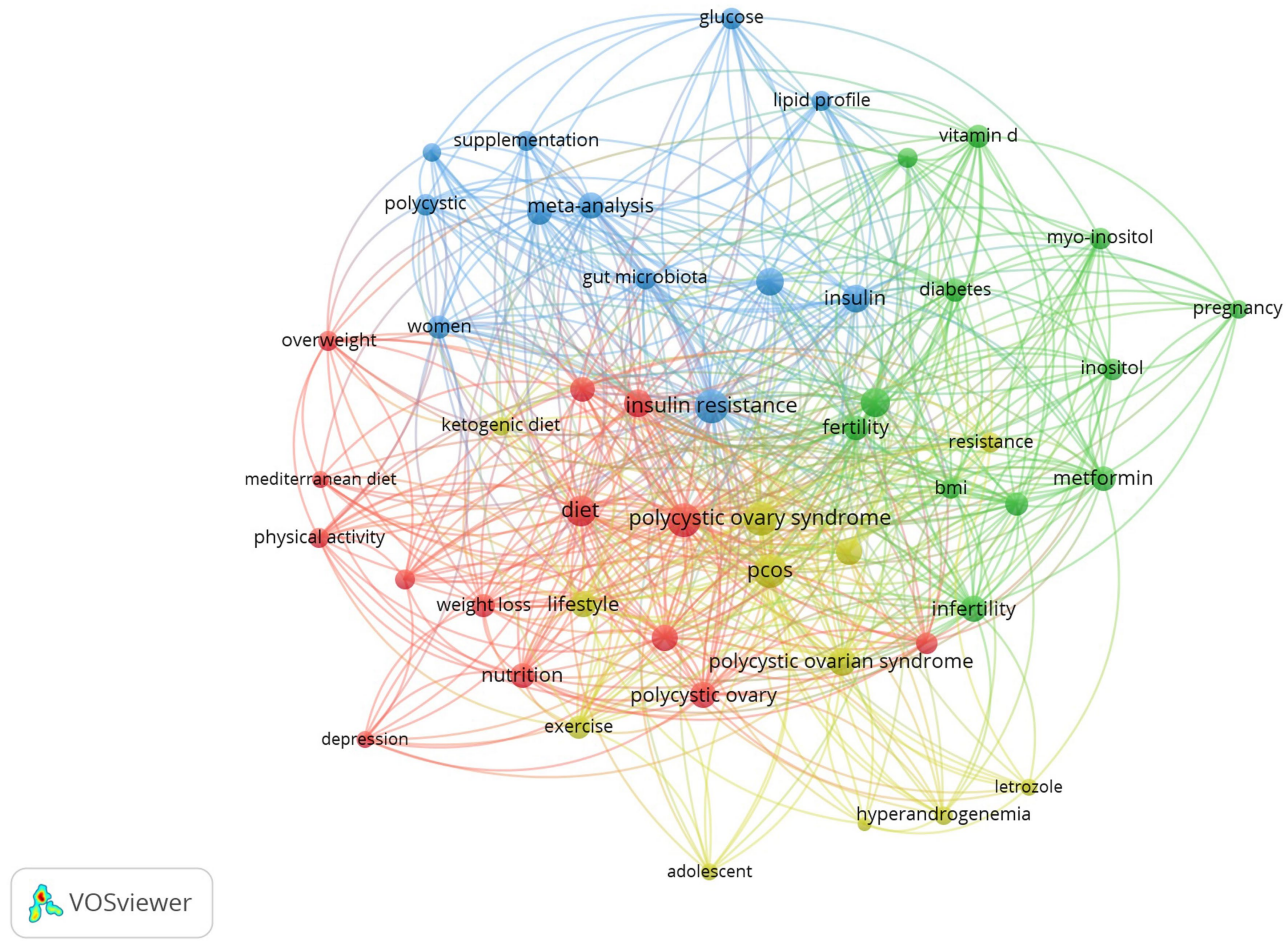
Keyword co-occurrence network visualization based on thematic clustering.

The co-occurrence analysis of the keywords revealed four large clusters, as shown in Figure 5, which illustrates the trends in PCOS and nutrition. The keyword co-occurrence analysis revealed four major thematic clusters: (1-red) lifestyle modifications and related complications in PCOS; (2-green) hormonal imbalances and fertility treatments; (3-blue) metabolic dysregulations; and (4-yellow) dietary intervention strategies.

**Figure 6:**
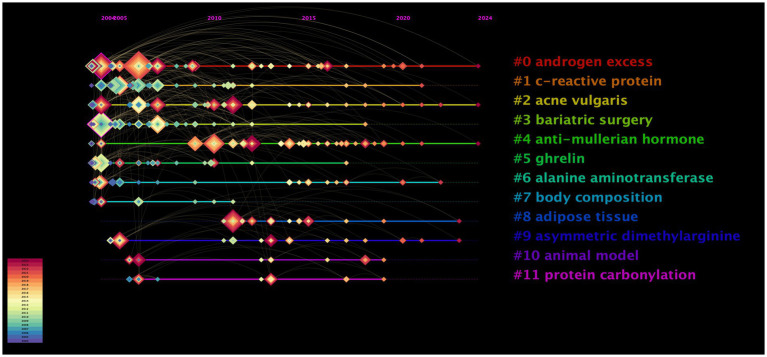
Timeline visualization of keywords related to nutrition and PCOS research. The colored diamonds along the horizontal lines represent keywords. The color of each diamond indicates the year of its occurrence, following the temporal scale (bottom left) from purple (2004) to dark red (2024). The size of the diamonds is proportional to the frequency of keyword appearances in that specific year, while the curved links represent co-occurrence relationships between topics.

The keyword timeline visualization in Figure 6 illustrates the progression of research, highlighting that keywords with rapid frequency increase over short periods to reveal emerging hotspots and frontier areas. Figure 6 visualizes the thematic progression of research over time. Horizontal lines represent distinct keyword clusters, while colored diamond-shaped nodes indicate keyword occurrences. The size of each diamond reflects the frequency of the keyword, and the color denotes the year (2004–2024), transitioning from cooler to warmer tones. The timeline suggests an evolution from foundational topics such as “androgen excess” toward more recent research frontiers, including “adipose tissue” and “protein carbonylation.” Over time, initial focus areas such as androgen excess, C-reactive protein, acne vulgaris, and body composition shifted toward adipose tissue, antimullerian hormone, and asymmetric dimethylarginine (Figure 6).

**Figure 7:**
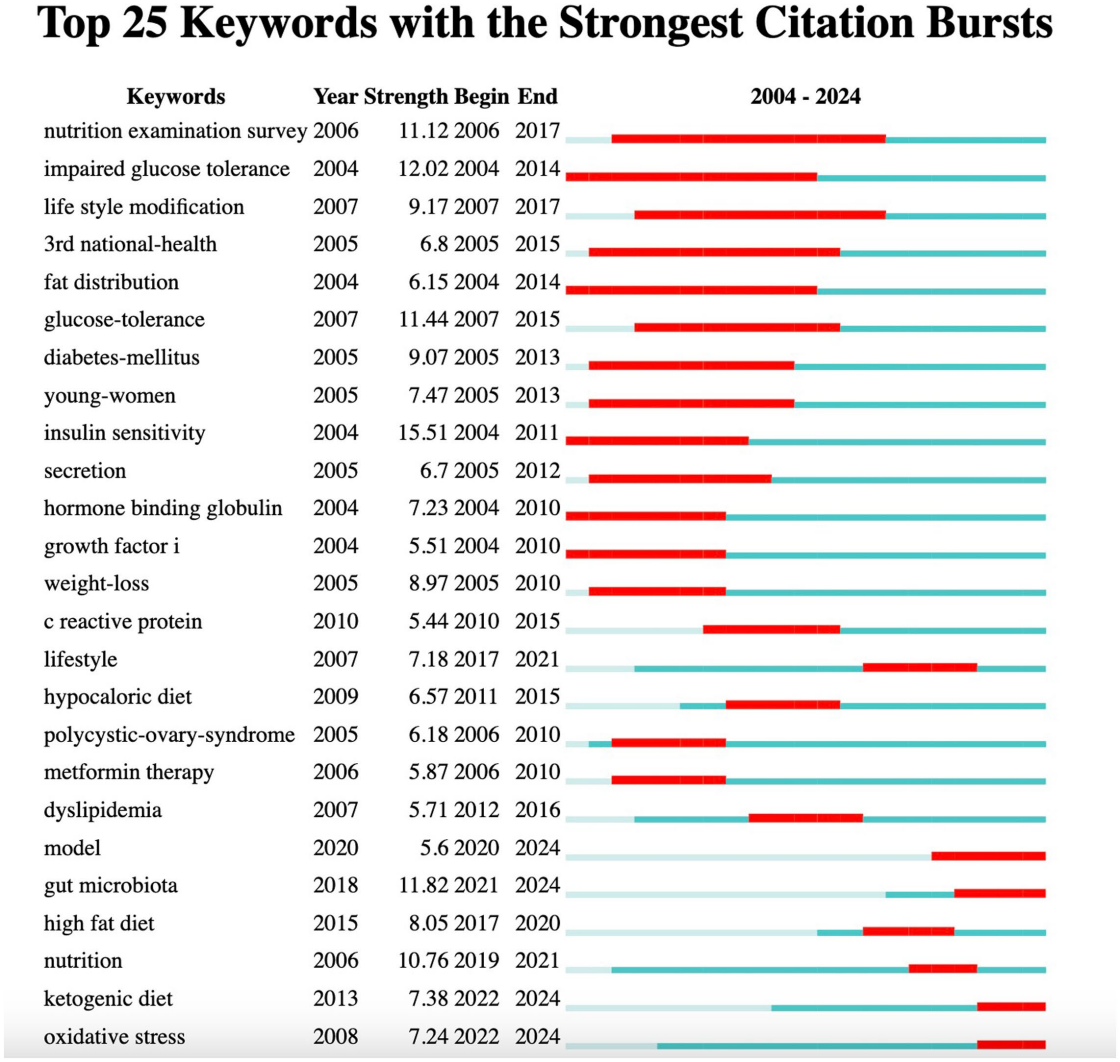
Keywords burst analysis of publications related to nutrition and PCOS.

The burst analysis of the 25 most frequently cited keywords is presented in Figure 7. From 2004--2024, 25 keywords with strong citation bursts in PCOS and nutrition research each have designated “start” and “end” years, marking their active periods in academic citations, with red indicating a sudden surge in citations during specific periods. Accordingly, the keywords with the greatest burst were “insulin sensitivity” (*n* = 15.51), followed by “impaired glucose tolerance” (*n* = 12.02) and “gut microbiota” (*n* = 11.82). The keyword “nutrition examination survey” had the longest burst period, from 2006–2017, followed by “impaired glucose tolerance” (2004–2014), “life style modification” (2007–2017), “3rd national health” (2005–2015), and “fat distribution” (2004–2014). As of 2024, the most frequently used keywords are “model,” “gut microbiota,” “ketogenic diet,” and “oxidative stress,” reflecting current research priorities and emerging trends. The evolution of keywords and the timing of citation bursts reflect shifts in research focus, transitioning from “metformin therapy” (2006–2010) and “weight loss” (2005–2010) to more recent topics such as the “ketogenic diet” and “oxidative stress.” This underscores the increasing focus on diverse dietary patterns, both in the context of PCOS and its associated complications (Figure 7).

## 4 Discussion

In this bibliometric study, a total of 1,248 publications related to nutrition and PCOS were retrieved from the WoS and Scopus databases for the period 2004–2024. These publications were contributed by 5,385 authors across 53 countries, with a notable international collaboration rate. Although there were fluctuations at the beginning, there was growing research interest in the fields of PCOS and nutrition over time. The trends of keywords revealed a progression from basic biological mechanisms to complex nutritional interventions and their impact on the management of PCOS. This bibliometric analysis is a scientifically valuable visual study for evaluating the position of the PCOS and nutrition theme within the literature, and it is also highly important in clearly identifying the thematic areas that future research can focus on. The most frequently cited journals and articles indicate that endocrine-related research constitutes a central focus within the field. Notably, there is a marked scholarly interest in myoinositol as a nutritional intervention strategy.

There is no geographical regional concentration in terms of the number of published articles. The United States has been found to be the most influential country because of the high number of articles it has published and the number of citations, and the difference with other countries in terms of the average number of citations it has received is quite striking. Italy is the second-largest country in terms of the number of influential publications, although Italy has fewer articles. Although Iran has a high volume of publications in the field, its comparatively lower citation count may reflect limitations in research visibility, international collaboration, or overall scientific impact. As previously emphasized, the results of this study reveal the necessity of conducting studies in low- to middle-income countries to reveal the relationship between nutrition and PCOS (27).

Nutrients appears to be the journal with the greatest contribution to nutrition-themed studies in the management of PCOS. In particular, its recent publications on the gut microbiota indicate that it follows current research trends (28, 29). However, when citation analyses are considered, the most influential journal is the Journal of Clinical Endocrinology & Metabolism, which more frequently addresses both endocrinological parameters and nutrition in the management of PCOS (30–32). The clinical guidelines published by Legro LS (25) bring this journal to the forefront, and the studies on omega-3 fatty acids in PCOS management published in this journal are also noteworthy (33, 34). In the field of PCOS and nutrition, the studies with the highest co-citation rates predominantly focus on consensus statements, clinical guidelines, and diet-based interventions. Common themes among these highly cited studies include nutritional interventions—particularly low glycemic index diets—and lifestyle modifications. The journal *Fertility and Sterility* has demonstrated its central role in this research domain by publishing the three most frequently co-cited articles (35–37), alongside significant contributions from Moran LJ.

The co-citation network analysis conducted in the present study demonstrated which publications have gained prominence in the literature and how they are interrelated. The studies published by Teede HJ, Escobar-Morreale HF, and Moran LJ, who are part of the Teede HJ research team, have received a high number of co-citations and occupy a central position within the literature. This finding indicates that these publications are regarded as fundamental reference sources in the field.

The network map obtained from the keyword co-occurrence analysis reveals that scientific production in the field of PCOS is concentrated around four distinct thematic clusters. These clusters distinctly represent the research orientations in the field, both in terms of subject matter and conceptual content. The red cluster, which encompasses lifestyle interventions, demonstrates that PCOS is not only a hormonal or reproductive disorder but also a chronic condition managed through lifestyle and behavioral approaches (38, 39). The Mediterranean diet is an anti-inflammatory dietary model rich in complex carbohydrates, fiber, and monounsaturated fats. A newly reported direct relationship has been identified between adherence to the Mediterranean diet and the clinical severity of the disease in women with PCOS. This relationship supports the therapeutic role of foods and nutrients in the Mediterranean dietary pattern in terms of adiposity, inflammation, insulin resistance, and hyperandrogenaemia—key factors in the pathogenesis of PCOS (40). The inclusion of psychosocial parameters (e.g., depression and quality of life) indicates the adoption of a biopsychosocial model in this area. The green cluster represents the clinical dimensions of studies related to hormonal imbalances and infertility in patients with PCOS. In this cluster, inositol and vitamin D appear to be complementary treatment options (41, 42). However, the evidence-based guidelines published by Teede and colleagues (1) in 2023, which play a significant role in the evaluation and management of PCOS, state that evidence for inositol is insufficient and does not include vitamin D. The blue cluster focuses on the biochemical, molecular, and metabolic foundations of PCOS. Keywords such as “systematic review” and “meta-analysis” also appear in this cluster, indicating that the studies in this group are based on high levels of evidence. This cluster reflects scientific efforts aimed at understanding the underlying pathophysiological processes of PCOS, such as the gut microbiota and oxidative stress, rather than just its symptoms (43, 44). Finally, the yellow cluster specifically represents animal studies (e.g., letrozole, high-fat diet), and more limited studies have been conducted during adolescence. In animal models, letrozole is used in combination with a high-fat diet to induce PCOS, as it can cause hypothalamic inflammatory reactions and lead to abnormal glucose and lipid metabolism (45). Additionally, studies have reported that energy-restricted and nutrient-dense ketogenic diets may be effective in managing PCOS in both adults and adolescents (46, 47). These thematic clusters show that research in the field of PCOS is expanding beyond clinical diagnosis and treatment to include lifestyle, molecular mechanisms, and integrated health management.

The citation burst analysis indicates a temporal change in research priorities within the field. The initial period (2004–2010) was characterized by a focus on metabolic and endocrine topics, including “insulin sensitivity” and “impaired glucose tolerance.” In contrast, in recent years (2020–2024), interest in dietary interventions and systemic factors, particularly the “gut microbiota,” “ketogenic diet,” and “oxidative stress,” has increased. The findings of this study reveal notable gaps regarding the inclusion of psychosocial variables, pediatric populations, and genetic factors. Addressing these gaps through more holistic and interdisciplinary approaches in future research may enhance the overall value and impact of the findings.

Hyperandrogenism, characterized by androgen excess and recognized as a major contributor to many complications observed in patients with PCOS (48), has maintained its significance over time and has shown continuity since the early 2000s. Studies focusing on bariatric surgery have gained prominence since 2012, reflecting a growing interest in surgical approaches to treating obesity in patients with PCOS. One of the most recent clusters is “protein carbonylation,” indicating an increased research focus in recent years on oxidative damage and molecular biomarkers.

The most important strength of this bibliometric analysis is its ability to systematically and objectively map research trends. The use of not only a single database (WoS and Scopus) but also widely accepted analysis tools such as RStudio, VOSviewer and CiteSpace are among the strengths of this study. This study has several limitations. Since only English-language publications were included, important studies published in other languages (such as Turkish or Chinese) may have been excluded. Notably, conducting a citation-based analysis does not reflect the clinical significance or quality of a study. Moreover, bibliometric analyses do not evaluate the methodological adequacy of the studies or the validity of their findings.

## 5 Conclusion

This bibliometric analysis systematically reveals the increasing scientific output, evolving thematic trends, and shifting research priorities in the field of PCOS and nutrition. While countries such as the United States and Italy have contributed substantially influential publications, the limited representation of low- and middle-income countries remains a notable gap and represents a critical barrier to the development of globally applicable and equitable health strategies.

Beyond a mere quantitative expansion, the findings highlight a clear paradigmatic shift in the literature over the past two decades. Research has transitioned from traditional approaches centered on weight management and insulin-related mechanisms toward more complex and systemic perspectives, including the gut microbiota-ovarian axis, oxidative stress, and metabolic interactions. This evolution reflects a growing recognition of PCOS as a multifaceted condition that extends beyond hormonal dysregulation alone.

In parallel, citation burst analyses indicate a marked increase in research attention toward the ketogenic diet in recent years (2020–2024). There remains a critical need for well-designed, long-term prospective studies and randomized controlled trials to evaluate not only the effectiveness but also the sustainability and cardiovascular safety of restrictive dietary approaches, particularly in populations already at increased cardiometabolic risk.

One of the major limitations identified in the current literature relates to study populations. Research in pediatric populations remains scarce, with existing studies predominantly focused on adult cohorts. Moreover, genetic factors and psychosocial determinants that may critically influence the onset and progression of the disease are largely overlooked. This imbalance underscores the need for future studies to prioritize early-life interventions that include pediatric populations, to account for psychosocial factors, and to integrate nutrigenetic approaches in order to better capture individual variability in treatment response.

Addressing these gaps will be essential for advancing the field toward more inclusive, personalized, and clinically relevant nutritional recommendations. Ultimately, advancing the field will depend on bridging the gap between expanding research output and the development of clinically actionable, personalized, and globally applicable nutritional strategies for PCOS management.

## Data Availability

The original contributions presented in the study are included in the article, further inquiries can be directed to the corresponding author.
